# Supplementary material for: ﻿Collective photothermal bending of flexible organic crystals modified with MXene-polymer multilayers as optical waveguide arrays
Source: Nat Commun. 2023 Jun 19;14:3627. doi: 10.1038/s41467-023-39162-5 (PMC10279756; doi:10.1038/s41467-023-39162-5)
Supplement: Supplementary file 1 — Supplementary Information﻿ [file 41467_2023_39162_MOESM1_ESM.pdf]

Supplementary Information

Collective photothermal bending of flexible organic crystals modified with MXene-polymer multilayers as optical waveguide arrays

Yang et al.

Supplementary Figures

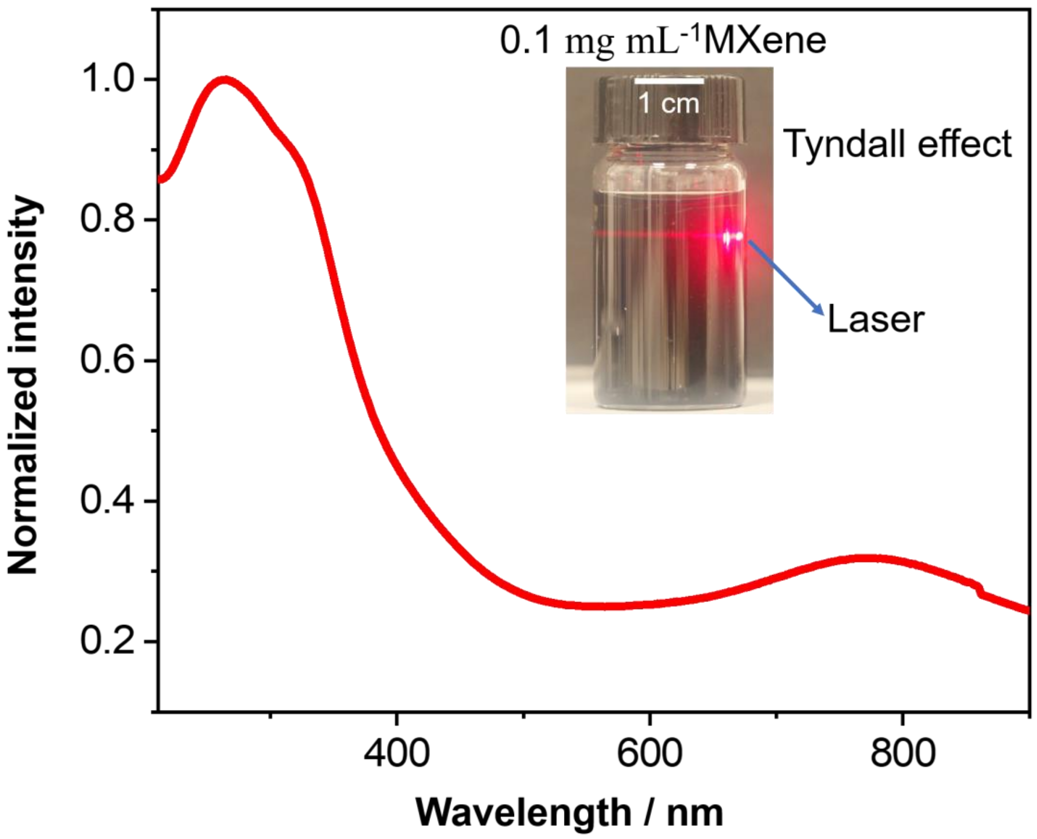

Supplementary Figure 1. UV-visible absorption spectrum. Plot of the absorption spectrum and the Tyndall effect (inset) of an aqueous suspension of MXene nanosheets (the source data is provided as a Source Data file).

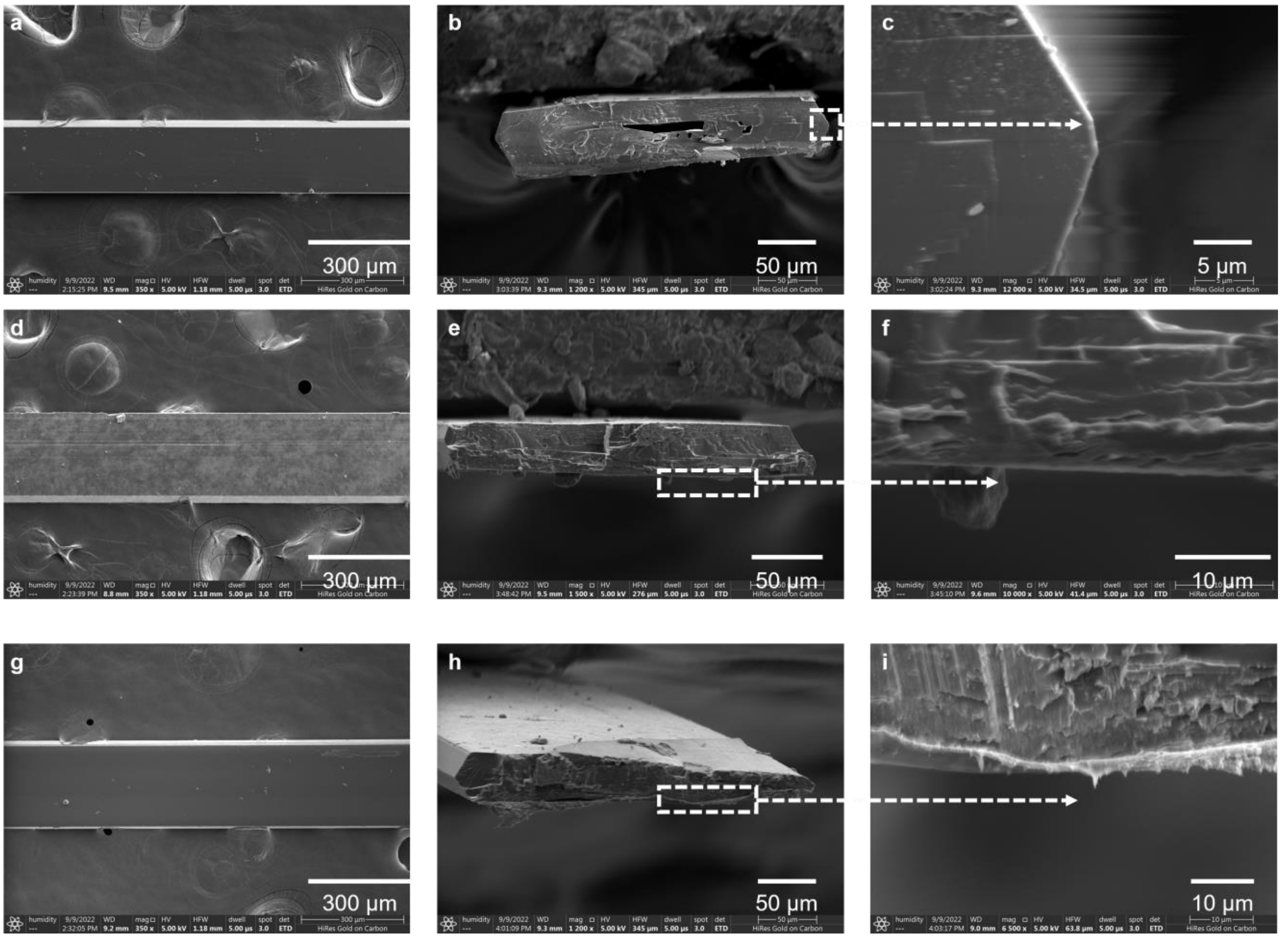

Supplementary Figure 2. Scanning electron micrographs of the hybrid crystals. (a,d,g) Images of **1**@P (a), **1**@P^2 (d), and **1**@P^3 (g). (b,c) Cross-sectional images of **1**@P. (e,f) Cross-sectional images of **1**@P^2. (h–k) Cross-section images of **1**@P^3.

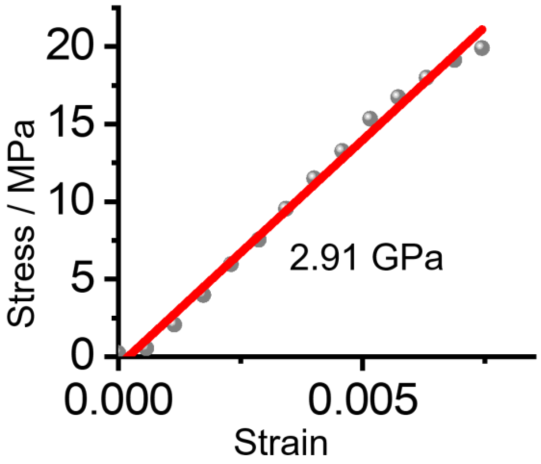

Supplementary Figure 3. Mechanical characterization of crystal of 1. The relevant section of the stress-strain profile of a crystal of **1** obtained by the three-point bending test is shown (the source data is provided as a Source Data file).

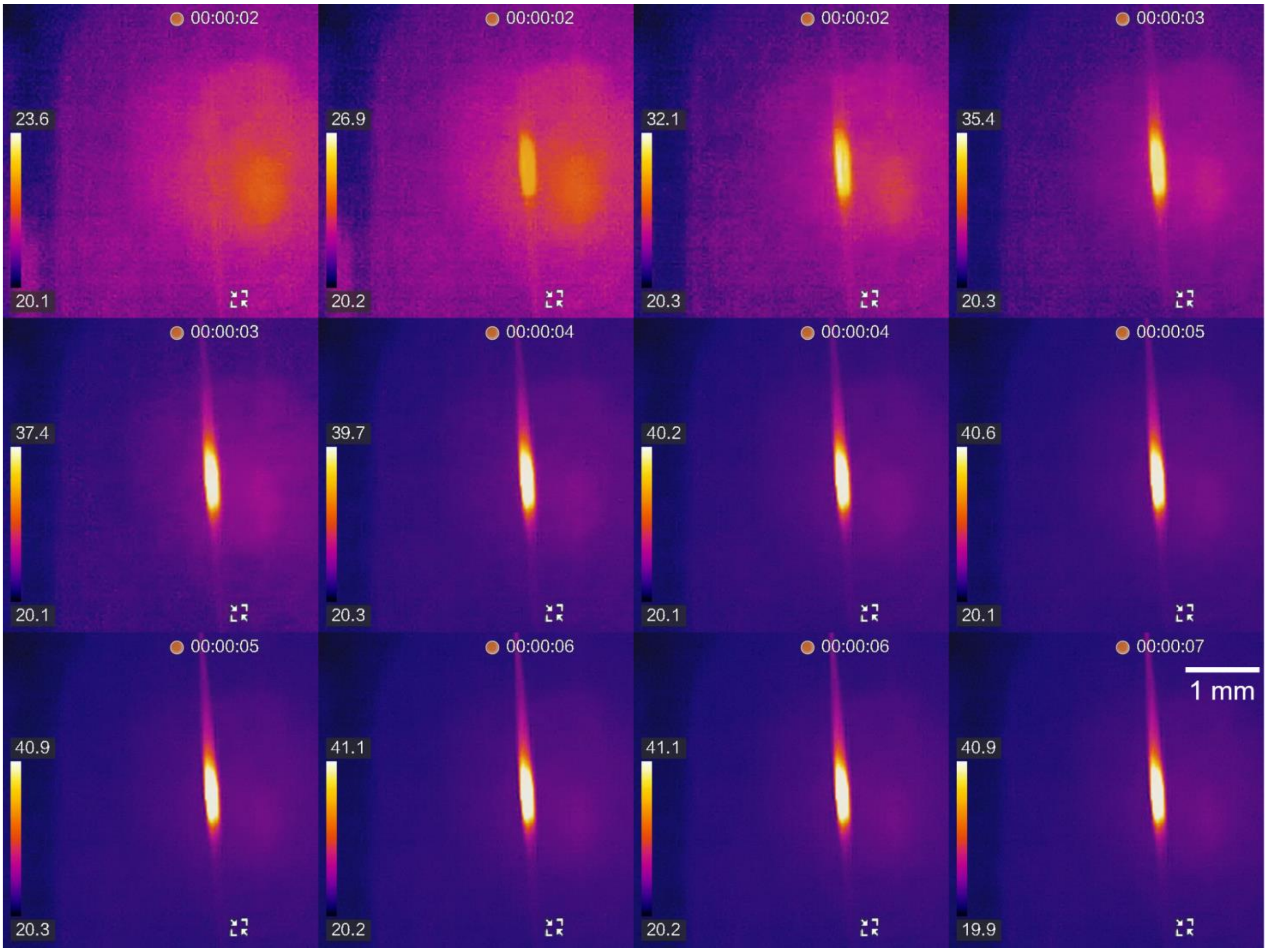

Supplementary Figure 4. Thermal imaging showing the photothermal conversion by a hybrid crystal. The temperature of the surface of a crystals of **1**@(PDDA/MXene)$_5$@PDDA/PSS is shown over time. The images are recorded at 0.5 s intervals from left to right and from top to bottom.

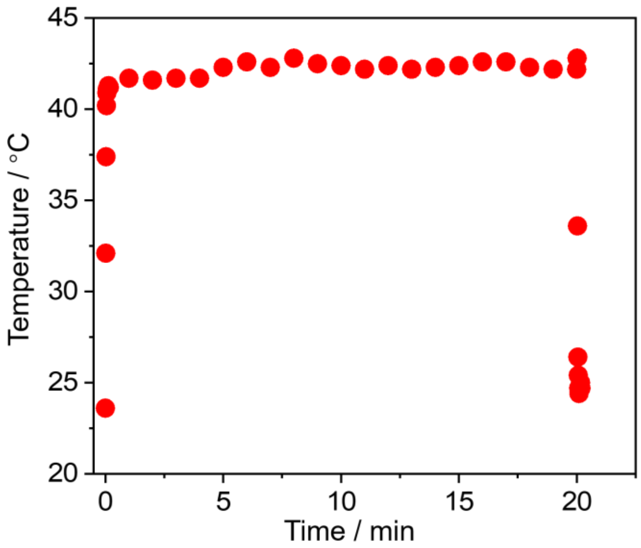

Supplementary Figure 5. Quantification of the photothermal conversion by the hybrid crystals. The temperature of the surface of **1**@(PDDA/MXene)$_5$@PDDA/PSS is plotted over time (the source data is provided as a Source Data file).

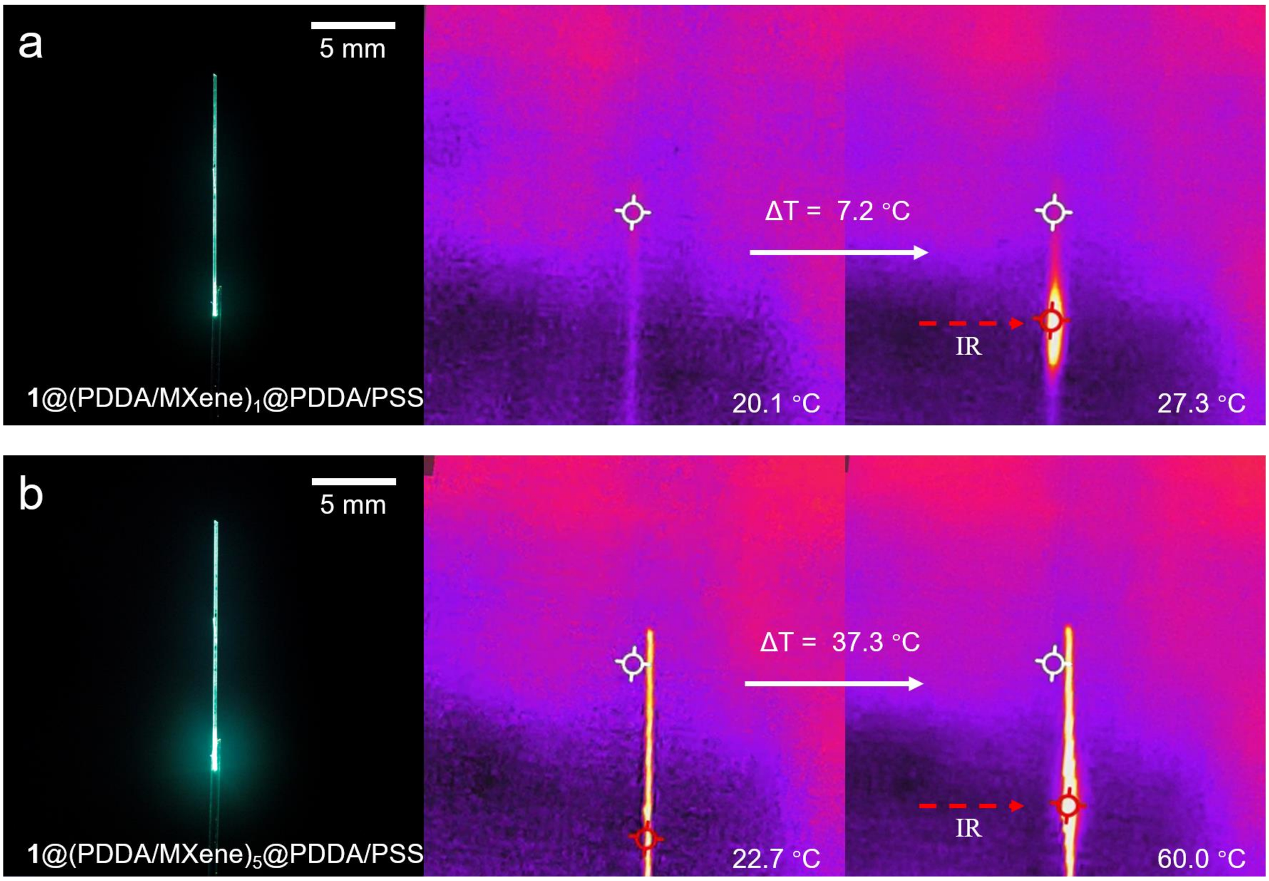

Supplementary Figure 6. Effect of the thickness of the MXene layer on the photothermal effect. The photographs (optical and thermal) illustrate the temperature change of the hybrid crystals having single layer (a) and five layers (b) of MXenes that have been excited with IR light under identical conditions (power, 408 mW).

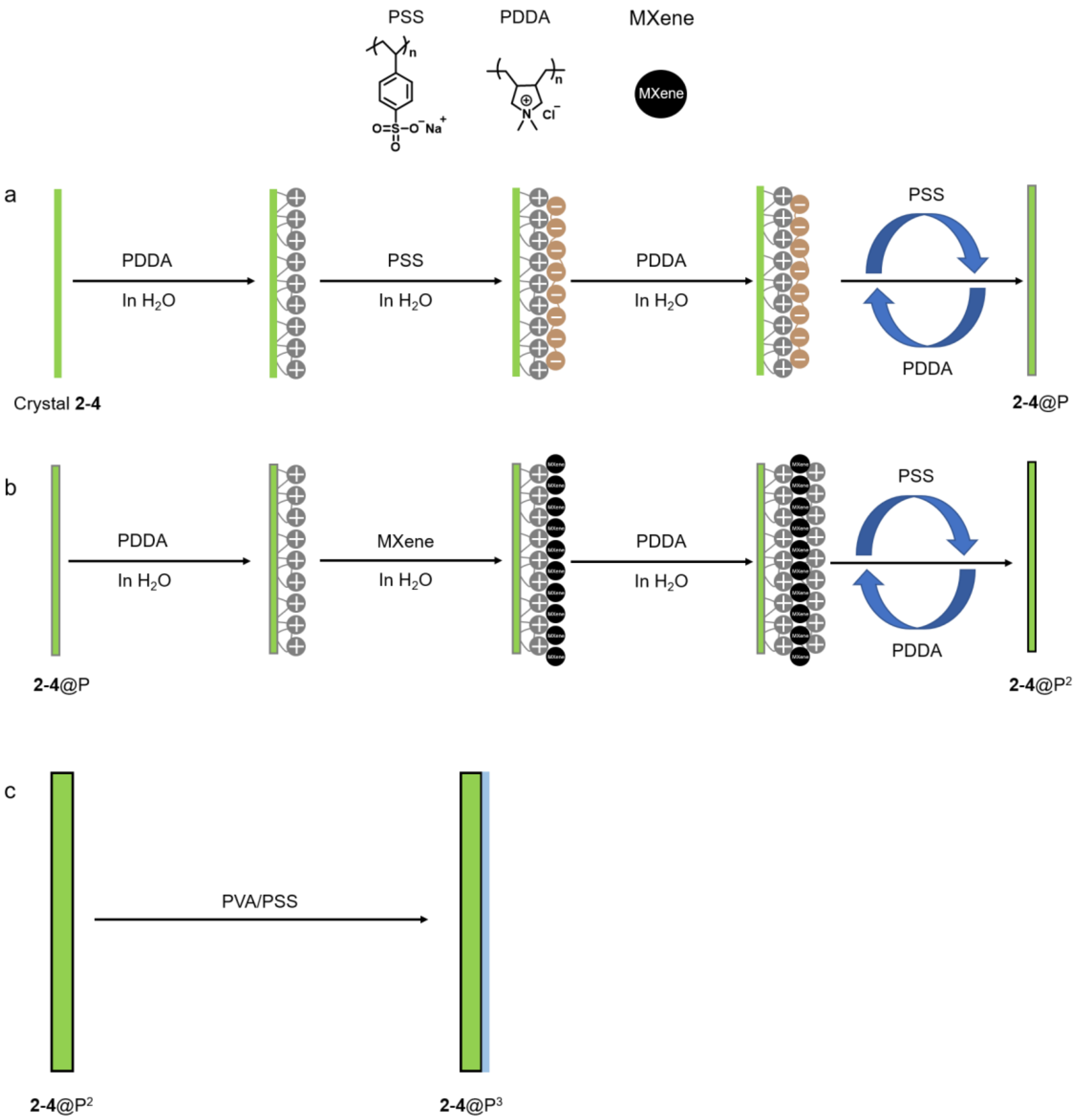

Supplementary Figure 7. A schematic showing the method used for preparation of the hybrid crystals. (a) Preparation of **2–4**@P. (b) Preparation of **2–4**@P^2. (c) Preparation of **2–4**@P^3.

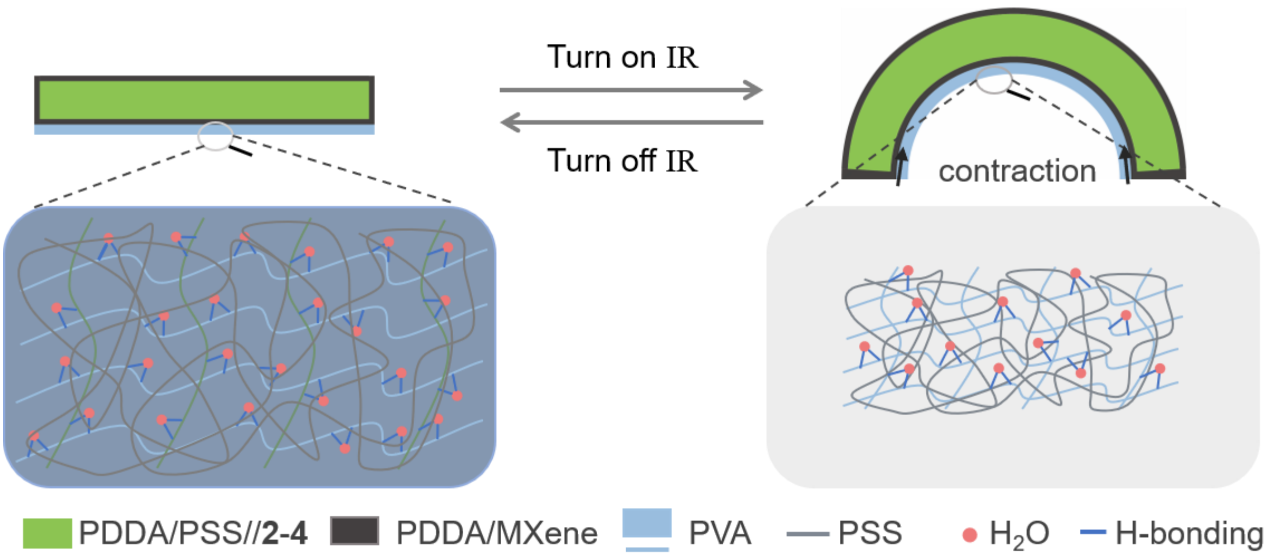

Supplementary Figure 8. Mechanism driving the bending of the hybrid crystals by photothermal effect. The diagram shows swelling or contraction of the polymer layer induced by heating by the MXene layer that is induced by exposure to infrared light.

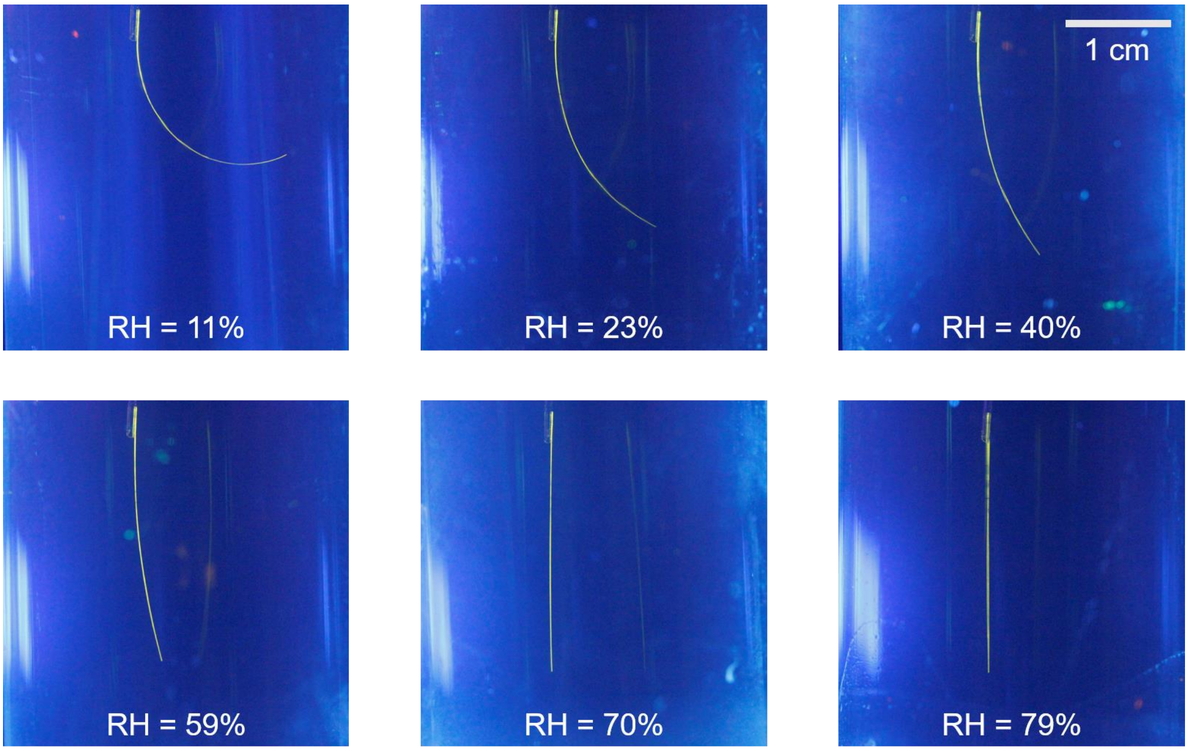

Supplementary Figure 9. Response of the hybrid organic crystals to humidity. Optical photographs are shown of **3**@P^3 that bends upon exposure to different aerial relative humidity (RH).

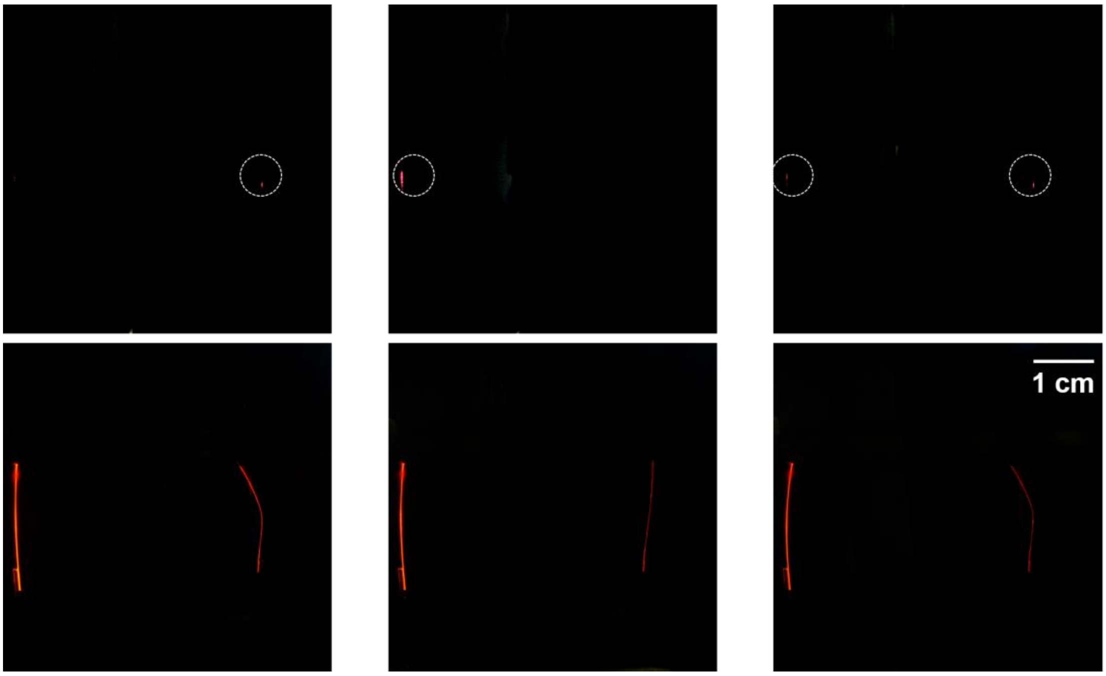

Supplementary Figure 10. Photographs of 4@PVA/PSS@PDDA/PSS (crystal on the left in each panel) and 4@P^3 (crystal on the right in each panel) under infrared light. The images on the top are recorded under daylight, while those on the bottom are recorded under UV light for better contrast. The white dotted circles indicate the point of excitation with infrared light.

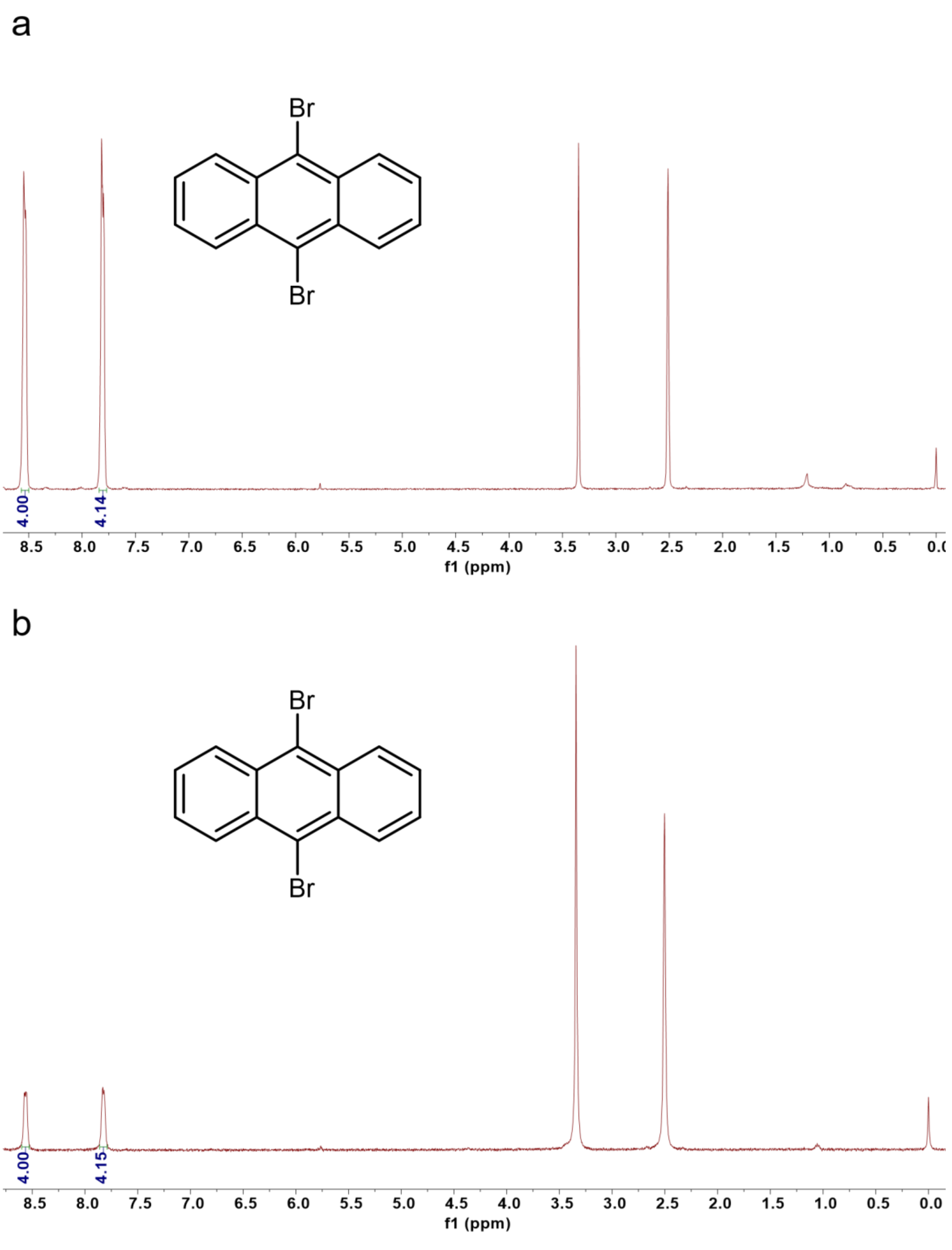

Supplementary Figure 11. ^{1}H NMR spectrum of compound 2 (DMSO-*d*6, 400 MHz). (a) Spectrum of compound **2** before heating. (b) Spectrum of compound **2** after heating at 100 ºC for 1 h.

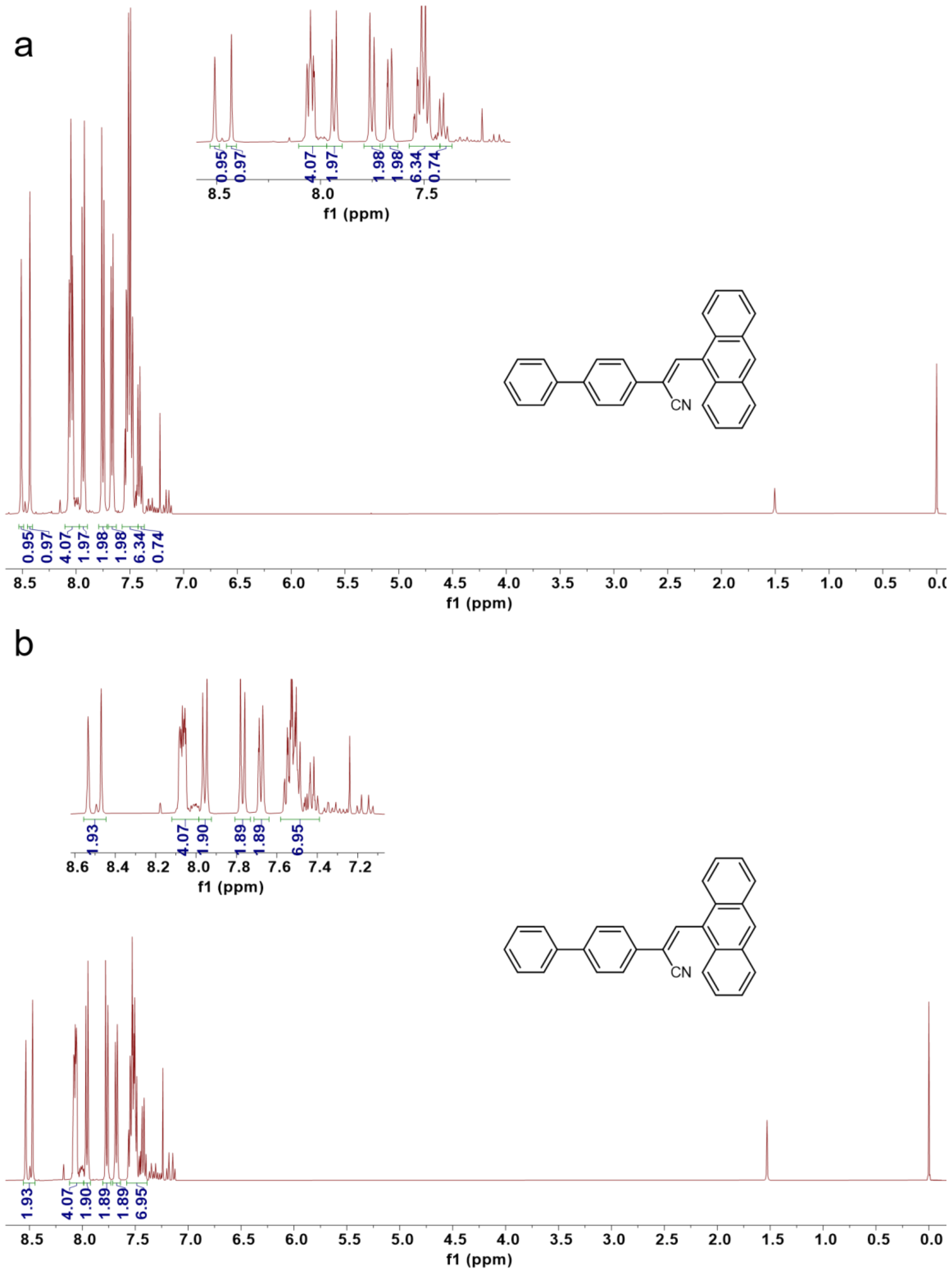

Supplementary Figure 12. ^{1}H NMR spectrum of compound 3 (DMSO-*d*$_6$, 400 MHz). (a) Spectrum of compound **3** before heating. (b) Spectrum of compound **3** after heating at 100 ºC for 1 h.

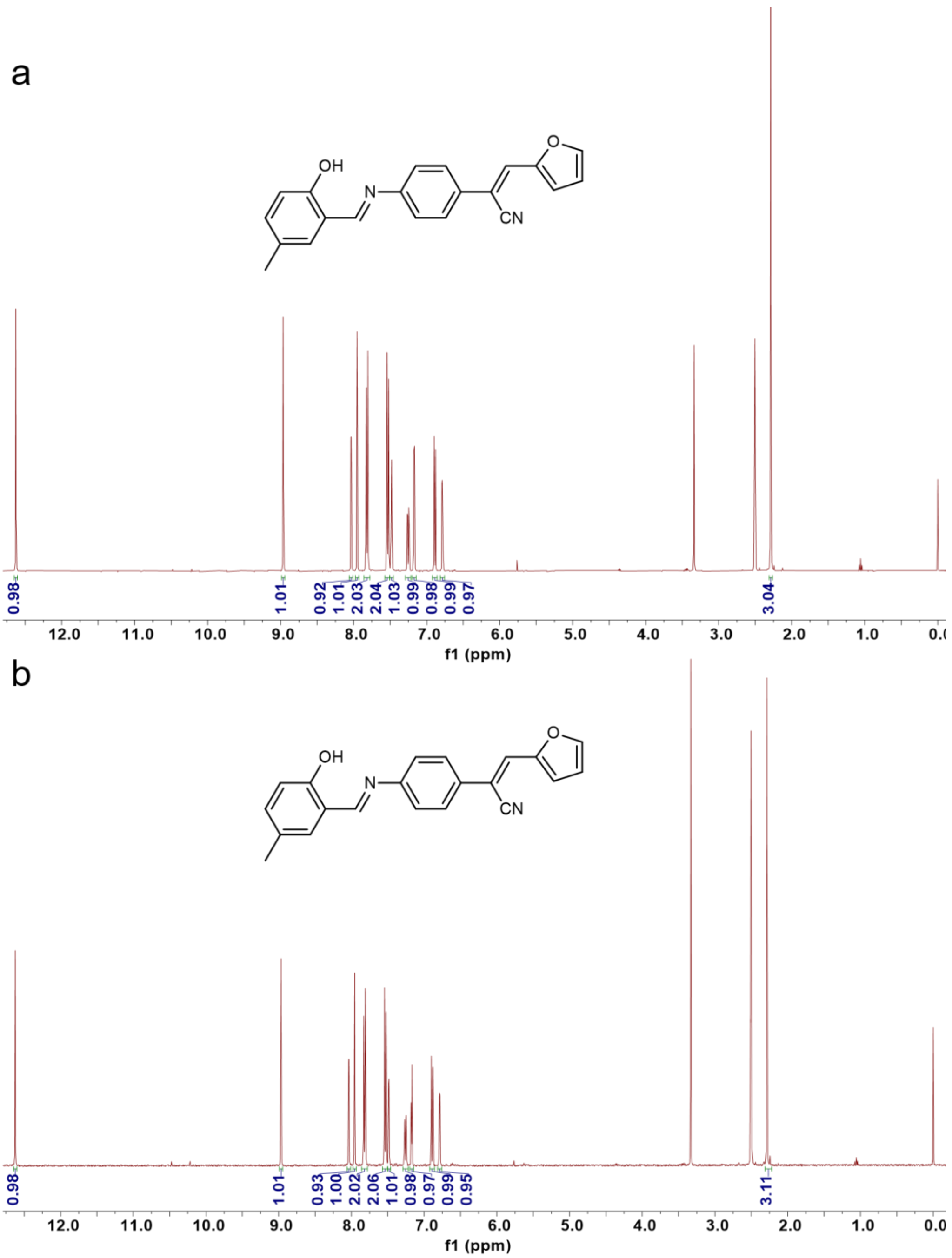

Supplementary Figure 13. ^{1}H NMR spectrum of compound 4 (DMSO-*d*$_6$, 400 MHz). (a) Spectrum of compound **4** before heating. (b) Spectrum of compound **4** after heating at 100 ºC for 1 h.

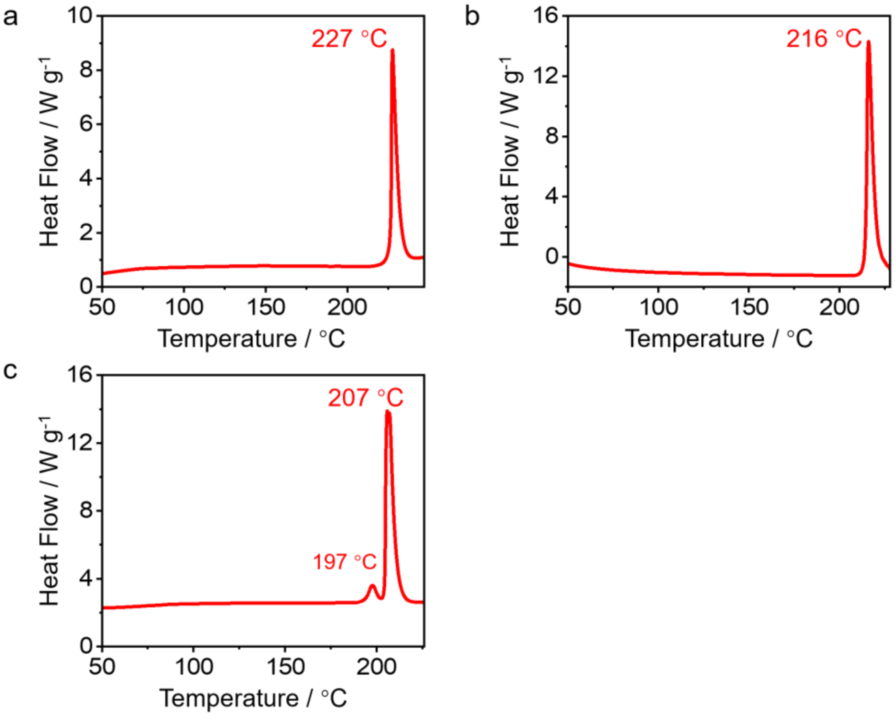

Supplementary Figure 14. Differential Scanning Calorimetric (DSC) analysis of the pure crystals. DSC analysis of crystals of **2** (a), **3** (b), and **4** (c) recorded at a heating rate of 30 K min^{-1}. The endothermic direction is up (source data is provided as a Source Data file).

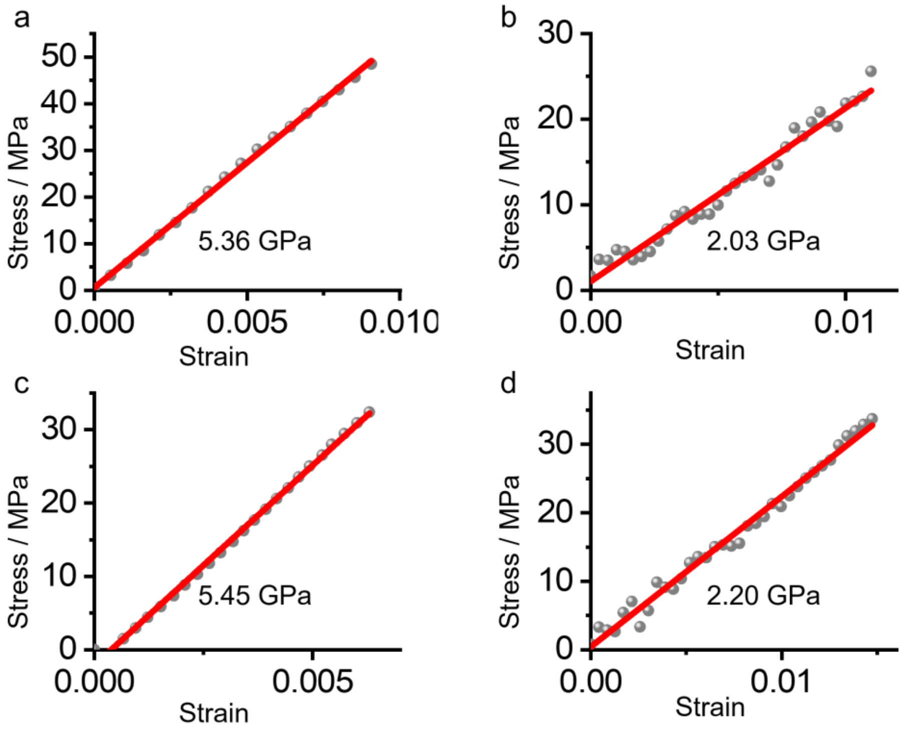

Supplementary Figure 15. Stress-strain profiles of the native and coated crystals determined by the three-point bending test. The relevant parts of the curves are shown for crystal of **2** (a), a hybrid crystal **2**@P^3 (b), crystal of **3** (c) and a hybrid crystal **3**@P^3 (d) (the source data is provided as a Source Data file).

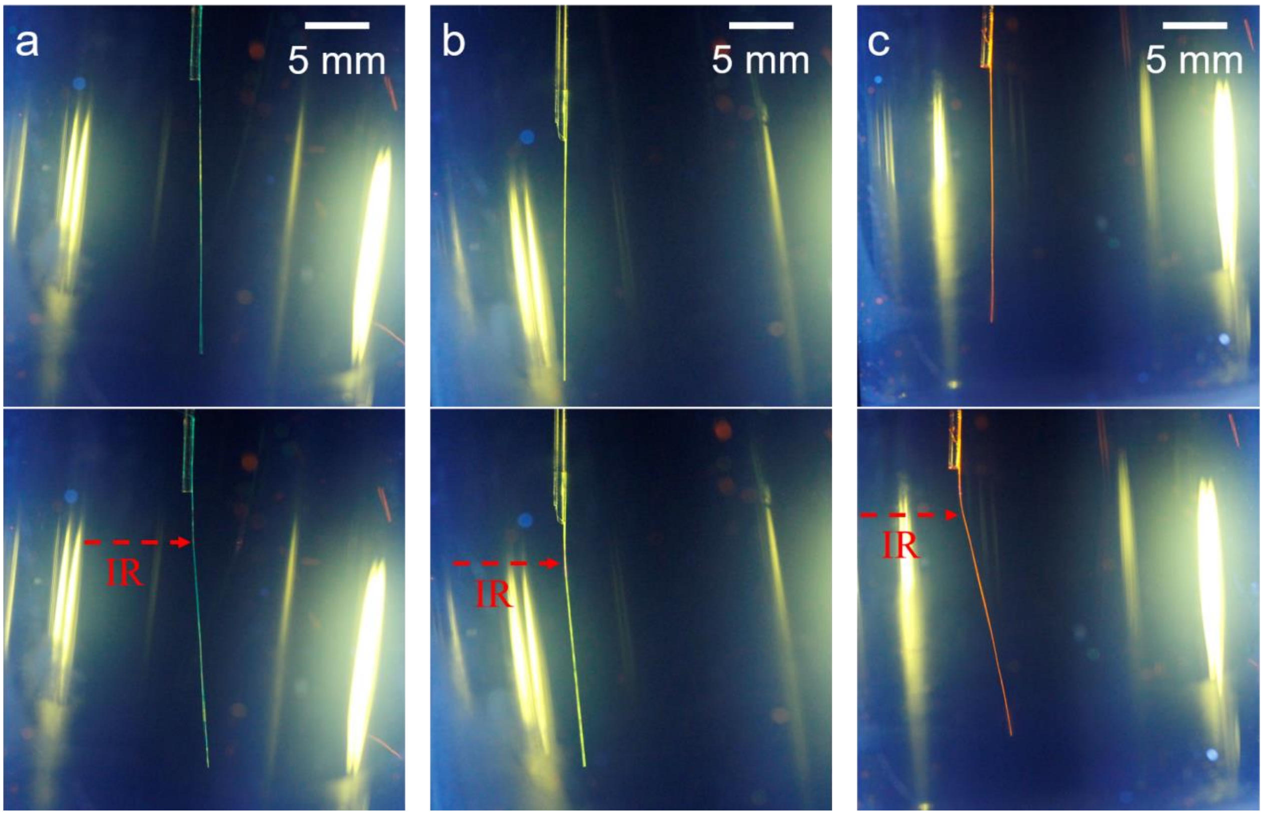

Supplementary Figure 16. Effect of the structure of the organic crystal on the mechanical response of the hybrid crystal. Photographs are shown of crystals **2**@P^3 (a), **3**@P^3 (b), and **4**@P^3 (c) exposed to infrared light before (top) and after exposure (bottom).

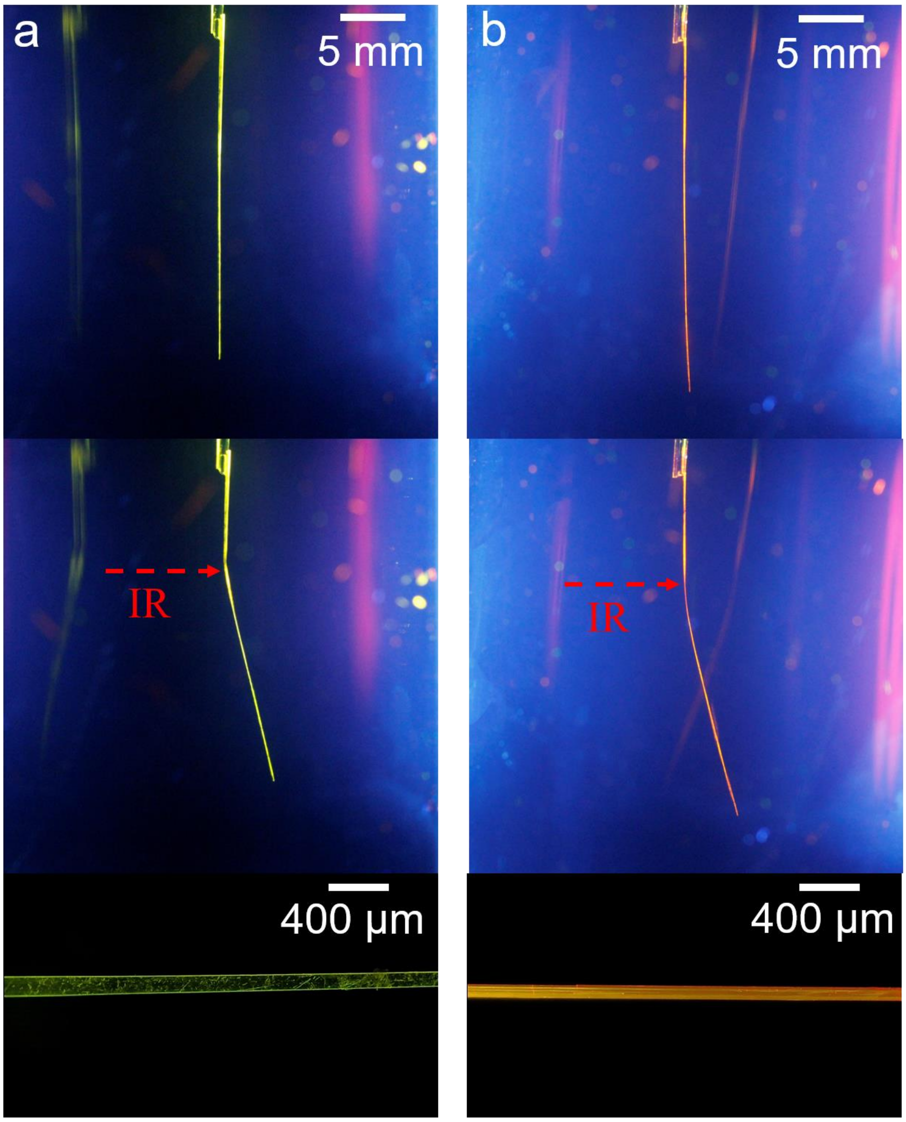

Supplementary Figure 17. The effect of crystal quality on the mechanical response of the hybrid crystals. Photograph are shown of crystals **3**@P^3 (a) and **4**@P^3 (b) that bend under infrared light before (top) and after exposure (bottom).

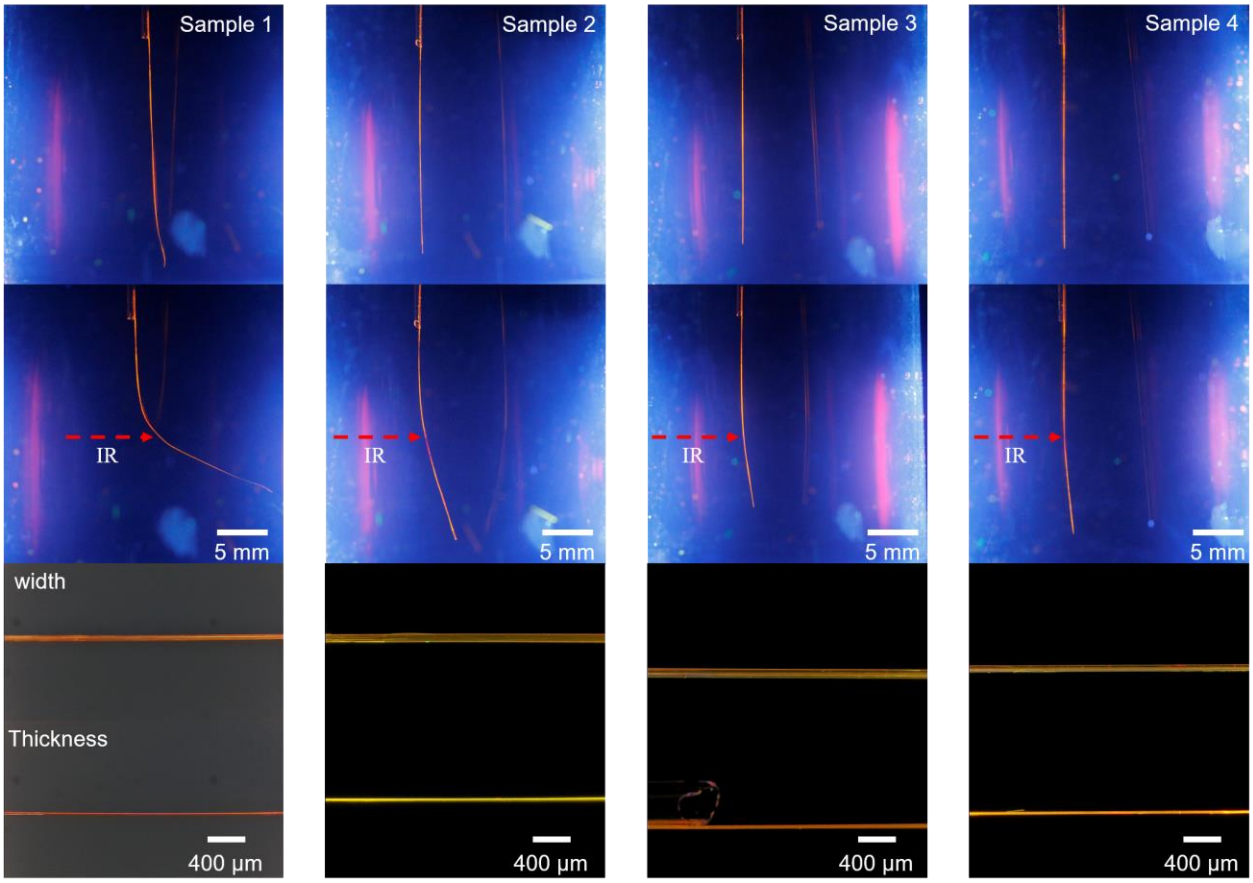

Supplementary Figure 18. Bending of hybrid crystals exposed to infrared light. Photographs of different sizes of crystal **4**@P^3 bending under infrared light (RH = 79%; power, 184 mW).

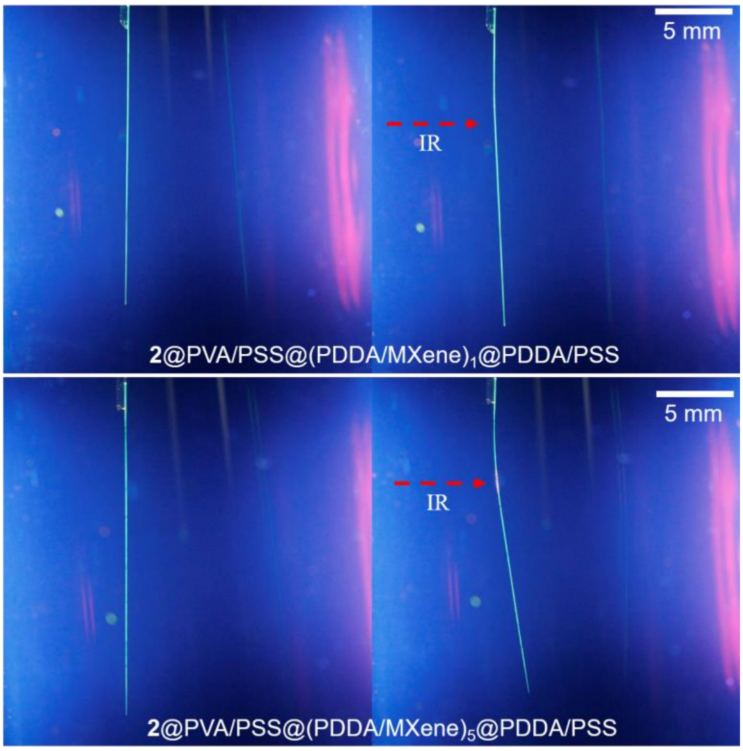

Supplementary Figure 19. Photothermal effect of the hybrid crystals. Photographs of hybrid crystals before and after exposure to infrared light (RH = 79%; power, 296 mW).

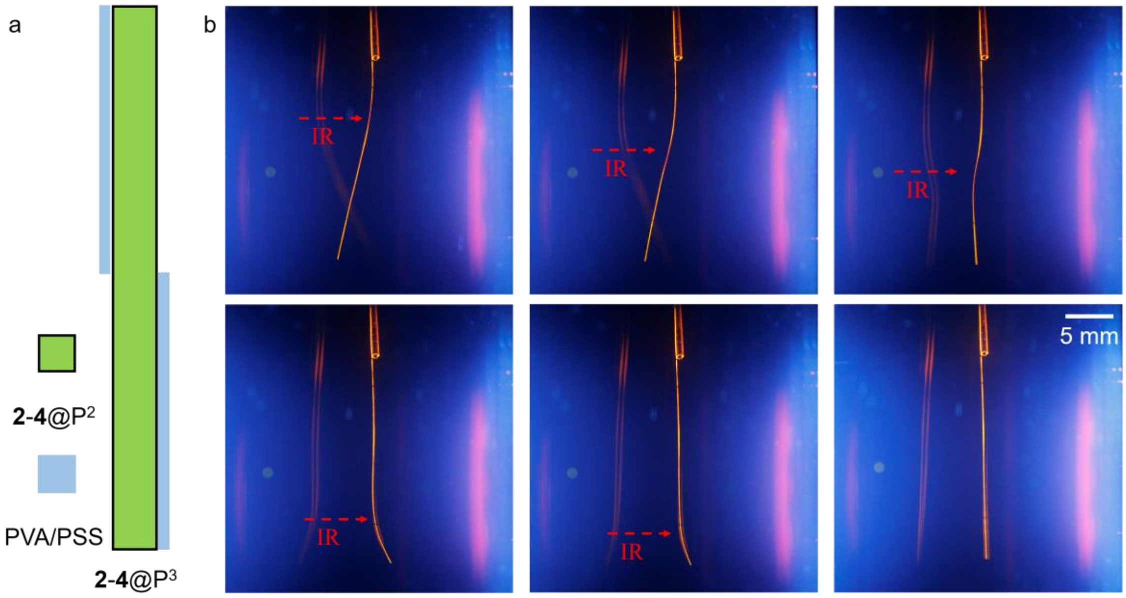

Supplementary Figure 20. Bidirectional bending of the hybrid organic crystals. (a) A schematic describing the method for deposition of PVA/PSS. (b) Photographs of **4**@P^3 bending in different directions under infrared light (RH = 79%).

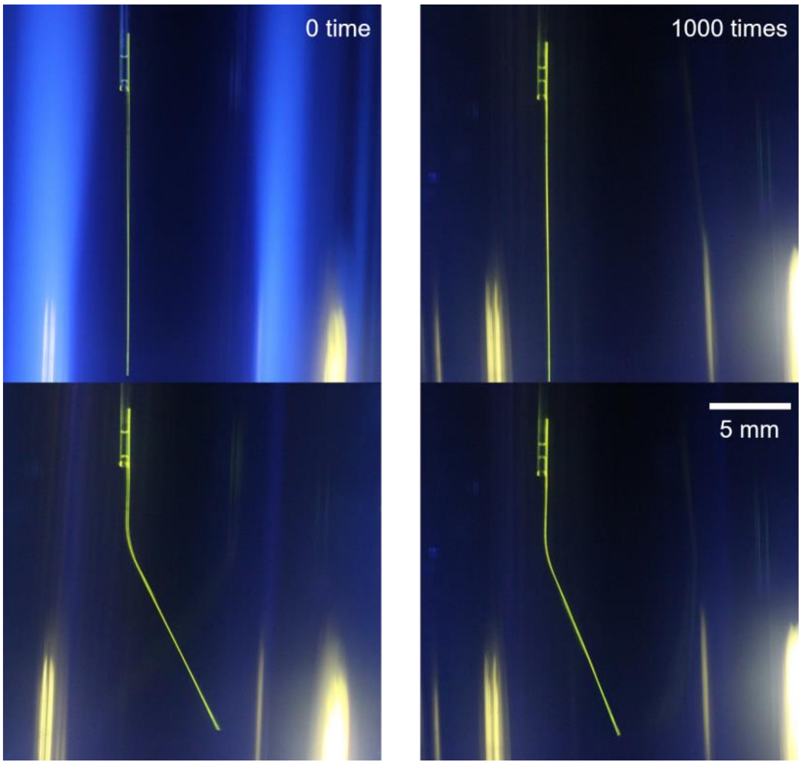

Supplementary Figure 21. Cyclability of the bending of a hybrid crystal. Photographs of **3**@P^3 are shown before (top) and after 1000 cycles of bending (RH = 79%; power, 184 mW).

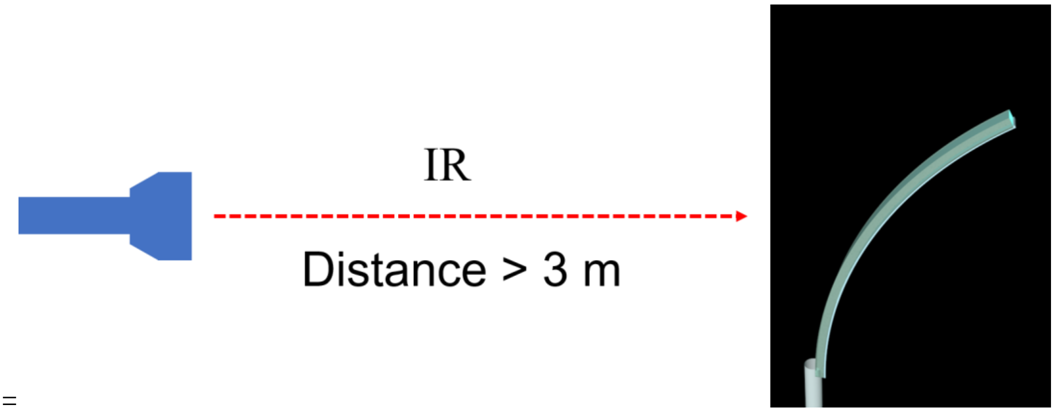

=

Supplementary Figure 22. Bending of hybrid crystal upon excitation over long distances. The image on the right shows a hybrid crystal that has been bent by exposure to infrared light where the light source was at a distance more than 3 meters away from the crystal.

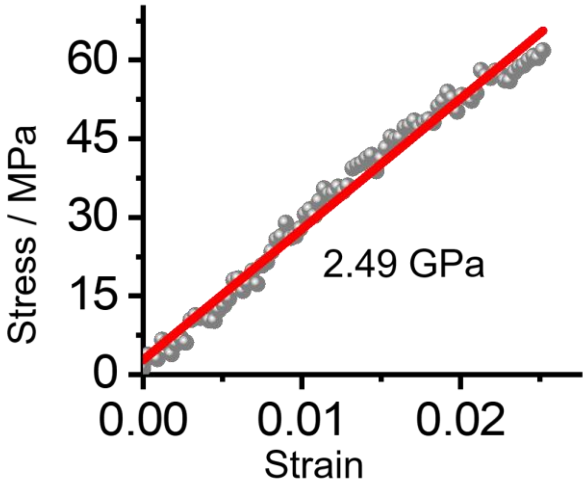

Supplementary Figure 23. Mechanical characterization of 5. Stress-strain profile of a crystal of **5** obtained by three-point bending test (source data is provided as a Source Data file).

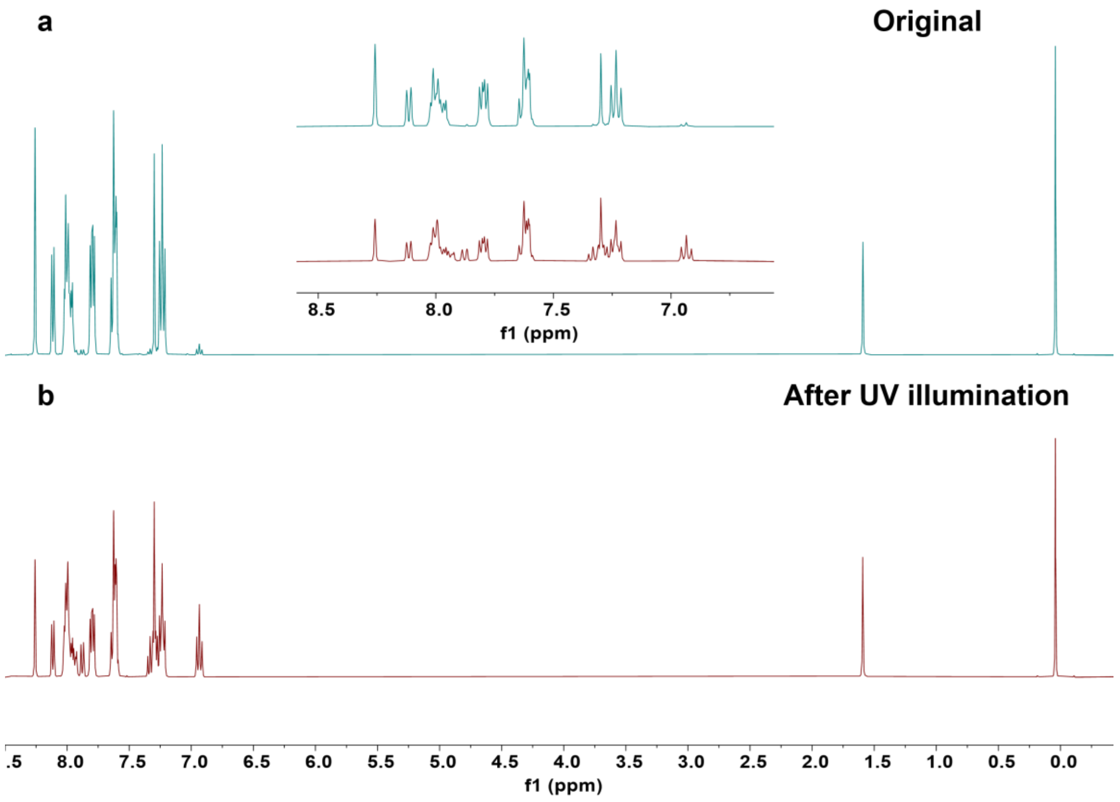

Supplementary Figure 24. ^{1}H NMR spectrum of compound 5 (chloroform-d, 400 MHz). (a) Spectrum of **5** before irradiation. (b) Spectrum of **5** after UV irradiation.

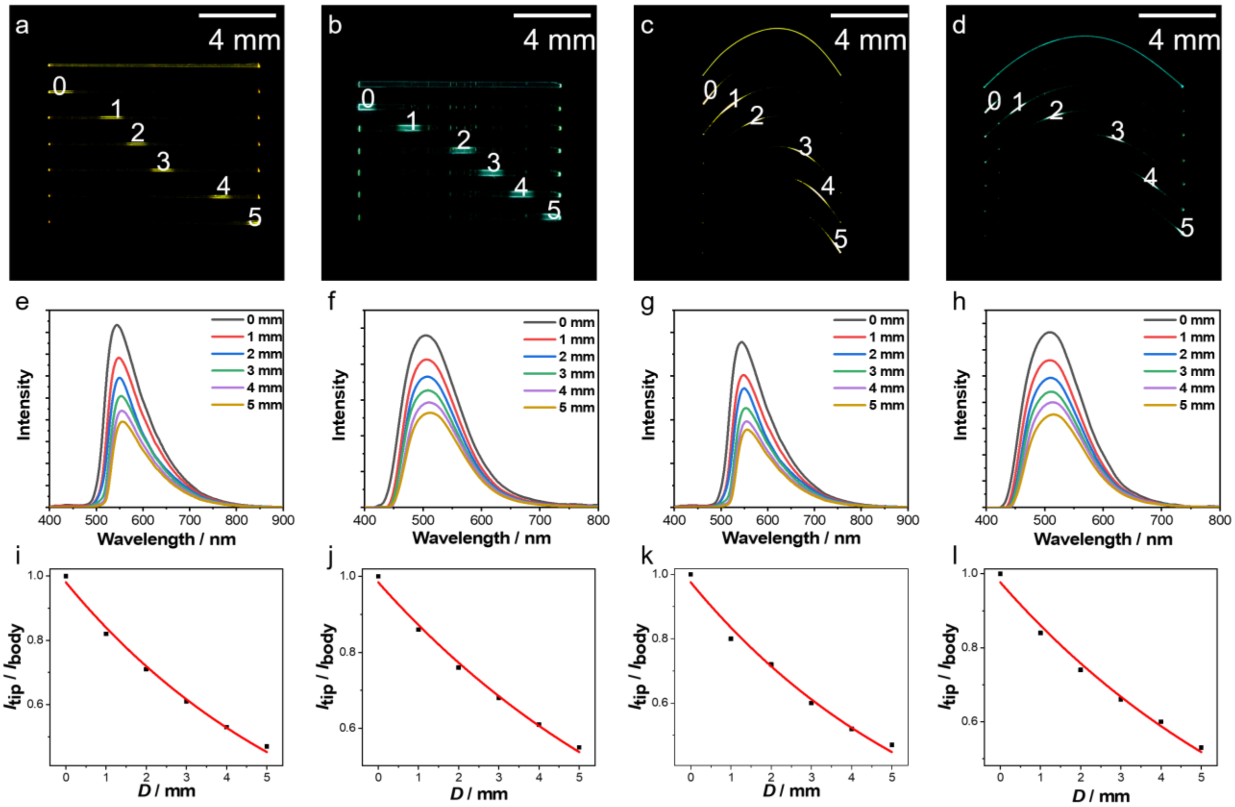

Supplementary Figure 25. Characterization of the optical waveguiding properties. (a–d) Image of **3**,**5**@P[3] used as a waveguide: **3**@P[3] in straight state (a), **5**@P[3] in straight state (b), **3**@P[3] in bent state (c), and **5**@P[3] in bent state (d). (e–h) Fluorescence spectra were collected at the fixed end of the crystal, while the crystals were exited at different position by 355 nm laser, position differences between the fixed end and the excitation position is defined as distance (mm). The panels e, f, g, and h correspond to the crystals shown in panels a, b, c, and d, respectively. (i–l) Decay of intensity with distance I_{tip}/I_{body}. The optical loss coefficient (α) was obtained by a single exponential fitting function. $I_{tip}/I_{body} = A\exp(-\alpha D)$, in which I_{tip} and I_{body} are the fluorescence intensities measured at the fixed end and the excitation position, respectively. A is the optical loss coefficient and D is position differences between the fixed end and the excitation position. The panels show **3**@P[3] in a straight state (i), **5**@P[3] in a straight state (j), **3**@P[3] in a bent state (k), and **5**@P[3] in a bent state (k). (source data is provided as a Source Data file).

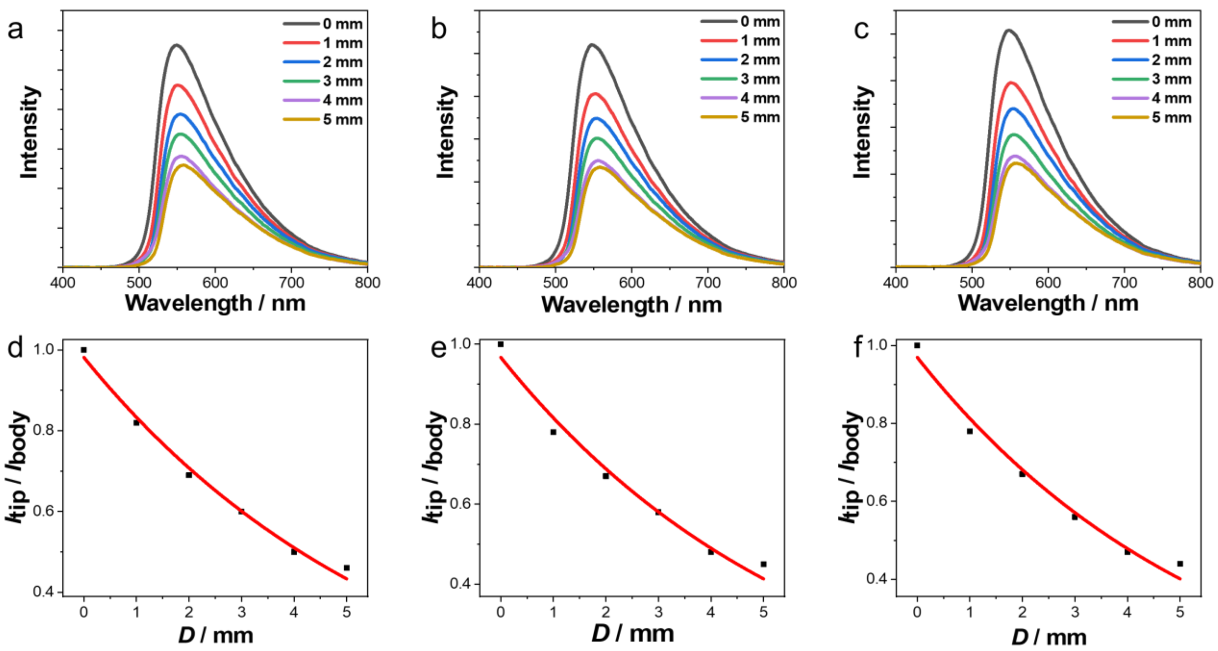

Supplementary Figure 26. Dependence of the optical loss on the crystal bending cycles. (a–c) Fluorescence spectra were collected at the fixed end of the crystal, while the crystals were excited at different position by 355 nm laser (10 Hz, 10 ns). The difference in position between the fixed end and the excitation position is defined as distance (mm). Panels a, b, and c correspond to optical loss after 0, 50, 100-fold bending, respectively. (d–f) Decay of intensity with distance I_{tip}/I_{body}. The optical loss coefficient (α) was obtained by a single exponential fitting function $I_{tip}/I_{body} = A\exp(-\alpha D)$, where I_{tip} and I_{body} are the fluorescence intensities measured at the fixed end and the excitation position, respectively. A is the optical loss coefficient and D is the distance between the fixed end and the excitation position. The panels show **3**@P^3 after 0 (d), 50 (e), and 100 (f) bending cycles. (source data is provided as a Source Data file).

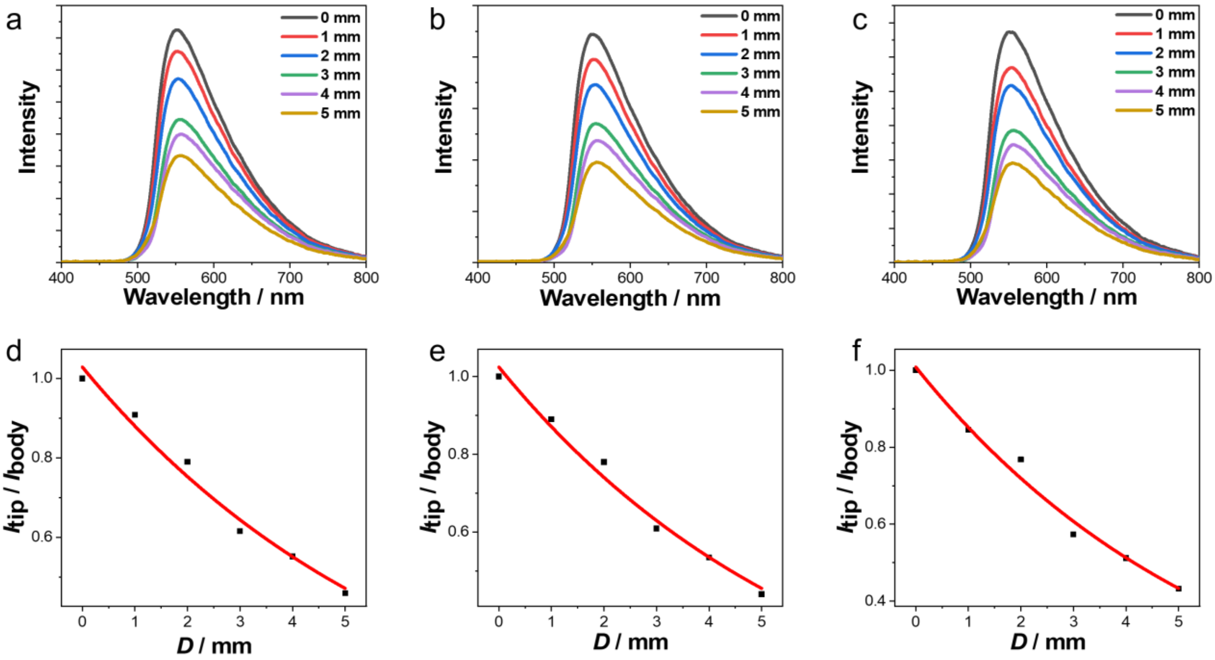

Supplementary Figure 27. Dependence of the optical loss on the duration of excitation. (a–c) Fluorescence spectra were collected at the fixed end of the crystal, while the crystals were excited at different position by a 355 nm laser (10 Hz, 10 ns). The difference in position between the fixed end and the excitation point is defined as distance (mm). Panels a, b, and c correspond to optical loss after excitation of 0 min, 30 min, and 60 min, respectively. (d–f) Decay of intensity with distance I_{tip}/I_{body}. The optical loss coefficient (α) was obtained by a single exponential fitting function $I_{tip}/I_{body} = A\exp(-\alpha D)$, where I_{tip} and I_{body} are the fluorescence intensities measured at the fixed end and the excitation position, respectively. A is the optical loss coefficient and D is position differences between the fixed end and the excitation position. The panels show **3**@P^3 at 0 min (d), at 30 min (e), and at 60 min (f) of irradiation. (source data is provided as a Source Data file).
