# Supplementary material for: ﻿Collective photothermal bending of flexible organic crystals modified with MXene-polymer multilayers as optical waveguide arrays
Source: Nat Commun. 2023 Jun 19;14:3627. doi: 10.1038/s41467-023-39162-5 (PMC10279756; doi:10.1038/s41467-023-39162-5)
Supplement: Supplementary file 3 — Description of Additional Supplementary Information [file 41467_2023_39162_MOESM3_ESM.docx]

**Description of Additional Supplementary Files**

Supplementary Movie 1.: Change in the surface temperature of **1**@(PDDA/MXene)_5_@PDDA/PSS over time.

Supplementary Movie 2.: Comparison of **4**@PVA/PSS@PDDA/PSS and **4**@P^3^ exposed to infrared light.

Supplementary Movie 3.: Collective bending of 3 × 3 arrays of crystals under infrared light.

Supplementary Movie 4.: Durability test of **3**@P^3^.

.

Supplementary Movie 5.: Durability test of **4**@P^3^.

.

Supplementary Movie 6.: Sensitivity test of the 1^st^ cycle of **3**@P^3^.

Supplementary Movie 7.: Sensitivity test of the 100^th^ cycle of **3**@P^3^.

Supplementary Movie 8.: Sensitivity test of the 1^st^ cycle of **4**@P^3^.

Supplementary Movie 9.: Sensitivity test of the 100^th^ cycle of **4**@P^3^.

Supplementary Movie 10.: Bending of **3**@P^3^ over 1000 cycles.

Supplementary Movie 11. Remote control of bending of **4**@P^3^.

.

Supplementary Movie 12.: Change of the bending angle of **3**@P^3^ over time.

Supplementary Movie 13.: Infrared and ultraviolet light driving **5**@P^3^ for optical signal transmission.

Supplementary Movie 14. Change of **3**@P^3^ optical signal output position over time under infrared light.
